# The thermodynamic cost of driving quantum systems by their boundaries

**DOI:** 10.1038/srep14873

**Published:** 2015-10-08

**Authors:** Felipe Barra

**Affiliations:** 1 Departamento de Física, Facultad de Ciencias Físicas y Matemáticas, Universidad de Chile, Santiago Chile

## Abstract

The laws of thermodynamics put limits to the efficiencies of thermal machines. Analogues of these laws are now established for quantum engines weakly and passively coupled to the environment providing a framework to find improvements to their performance. Systems whose interaction with the environment is actively controlled do not fall in that framework. Here we consider systems actively and locally coupled to the environment, evolving with a so-called boundary-driven Lindblad equation. Starting from a unitary description of the system plus the environment we simultaneously obtain the Lindblad equation and the appropriate expressions for heat, work and entropy-production of the system extending the framework for the analysis of new, and some already proposed, quantum heat engines. We illustrate our findings in spin 1/2 chains and explain why an XX chain coupled in this way to a single heat bath relaxes to thermodynamic-equilibrium while and XY chain does not. Additionally, we show that an XX chain coupled to a left and a right heat baths behaves as a quantum engine, a heater or refrigerator depending on the parameters, with efficiencies bounded by Carnot efficiencies.

Considerable experimental progress in various physical systems has been achieved toward the goal of controlling the dynamics of open quantum systems and their interactions with the environment^1^,^2^,^3^. For quantum computations or digital coherent quantum simulations, one may wish to have a system that is well isolated from the environment. For dissipative variants of quantum computations^4^ or creating new scenarios for non-equilibrium many-body systems, one would need to engineer the coupling to the environment. Recently, a setting in which the quantum system of interest interacts at its boundaries with an external quantum probe such that their coupling can be localized and can be switched on and off repeatedly with a controlled and well-defined state for the probe prior to the interaction has been experimentally realized^5^. This repeated interaction scheme has also been theoretically studied^6^,^7^. Importantly, the dynamics in an appropriate limit is a boundary-driven Lindblad equation. In this article, we explore the question of what is the thermodynamic cost of having such operations on an open quantum system and what are the thermodynamical quantities, such as heat and work that will determine the efficiency of quantum engines operating in this manner. Boundary-driven Lindblad equations have been intensively studied theoretically, particularly for one-dimensional quantum chains^7^,^8^,^9^,^10^,^11^,^12^,^13^,^14^,^15^,^16^,^17^,^18^,^19^, and powerful techniques have been developed to find their non-equilibrium steady states (NESS)^12^,^13^,^14^,^15^,^16^,^17^,^18^,^19^. These equations are also frequently used to describe quantum engines^20^,^21^,^22^,^23^ and other complex open quantum systems coupled to one or several environments^24^,^25^,^26^,^27^ because they are easy to implement. Nevertheless, a boundary driven Lindblad equation does not correctly describe a quantum system passively and weakly coupled to a heat-bath as often occurs in natural systems. It was pointed out recently^28^ that inconsistencies with the second law of thermodynamics may arise in this case and a careful examination of the coupling between a quantum refrigerator and the heat-baths^29^ reveals why boundary driven models are inappropriate for these situations. For a system passively and weakly coupled to one or several heat-baths the master equation derived in the Born-Markov-secular approximation^30^ yields a proper description of the system and the correct balance of heat flows and irreversible entropy production.

Thus, for our study, we consider explicitly the active (time-dependent) type of interaction between the system and the environment implemented in^5^ and the model developed in^6^,^7^. We apply the results of^31^,^32^ to derive the appropriate thermodynamical quantities and, in particular, we focus in the limit where the system is described by a boundary driven Lindblad equation.

Our main result is that driving at the boundaries, even though it looks like a work-free operation, actually might bring work to the system. We illustrate our findings on boundary-driven spin 1/2 chains coupled to one or two heat baths. We show that an XX spin 1/2 chain coupled in this way to a single heat bath relaxes to thermodynamic equilibrium while an XY does not because it is driven out of equilibrium by the power produced by the coupling to the heat bath. When two baths are connected to the chain, we observe that for different parameters, the chain operates as a quantum heat engine, refrigerator or heater, and we determine their efficiencies in the simple case of a chain of two sites. The rest of the paper is organized as follows. We start by reviewing first the thermodynamics of Markovian open quantum systems in the weak coupling limit^33^,^34^,^35^,^36^ and second a formulation^31^ where the “universe”, system plus the environment, evolves unitarily. After that, we consider the repeated interaction scenario for the system and the environment from which the boundary-driven Lindblad equation and the appropriate thermodynamical quantities for the open system are obtained. Then we illustrate our results in XX and XY spin 1/2 chains and offer our conclusions. Finally we have collected in section Methods some details of the calculations.

## Thermodynamics of open quantum systems

### Open system weakly and passively coupled to the environment

Let us briefly review the usual formulation of thermodynamics in open quantum systems^33^,^34^,^35^,^36^. Consider an open system described by a master equation in the Lindblad form


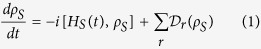


where the environment consists of several heat-baths *r* whose action on the system is represented by the dissipator


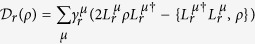


with 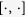 the commutator and 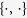 the anti-commutator. The operators 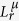 are system operators and represent the action of the environment over the system. When this equation is obtained from the weak coupling limit for a time independent system, one finds *global* Lindblad operators 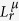 that are eigen-operator of the Hamiltonian *H*_*S*_^30^. For simplicity, we consider that the system can only exchange energy and no particles with the environment.

Now consider the internal energy 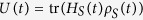 and the entropy 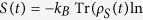

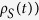
. The first law of thermodynamics 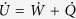 splits the rate of change of internal energy in two, power 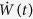 and heat flow 
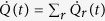
 with one contribution per heat-bath. For system passively and weakly coupled to the heat-baths, these quantities are defined as


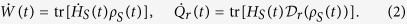


In section *Methods: Heat from a given reservoir in the weak-coupling limit* we justify these definitions. Note that if the Hamiltonian of the system is time independent, no work can be performed on the system and only heat is exchanged with the baths. In that case the system will typically reach a steady state. Consider now this to be the situation. The second law states the positivity of the entropy production 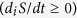, which is the difference between the time-derivative of the entropy 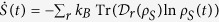 and the entropy flow from the environment to the system 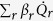,


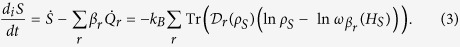


The canonical distribution 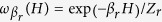 appears in the last equality of Eq. (3) due to the definition of heat that we plug in the first equality in Eq. (3). The second law 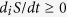 in Eq. (3) holds if for every *r*, 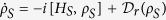 relaxes towards the unique equilibrium state 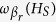. This is the local-detailed-balance condition^37^ i.e. if a single heat-bath is in contact with the system detailed balance as defined in^34^,^38^ holds. This property of the dissipators 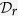 is satisfied in quantum master equations obtained in the weak-coupling and with the Born-Markov-secular approximation (global Lindblad equation). This framework has been applied successfully to the study of thermodynamic properties and efficiencies of engines^29^,^39^,^40^,^41^.

In boundary-driven systems the Lindblad operators 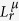 act locally on the boundaries of the system and in general the corresponding Lindblad equation does not satisfy local-detailed-balance. We come back to this point later. Following recent developments in the physics of non-equilibrium systems that have emphasized the importance of time reversal symmetry at the microscopic level of description^42^, a formulation of quantum thermodynamics in which the system plus the environment evolves unitarily has been proposed^31^. We consider this framework to analyze boundary driven systems.

### “universe” under unitary evolution

Let a system and an environment with Hamiltonians 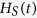 and *H*_*E*_ (time independent), respectively, coupled by an interaction potential *V*(*t*) evolve with total Hamiltonian 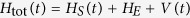. The environment might consists of several heat baths 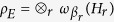 with 
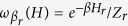
 the initial density matrix for the reservoir *r*. Initially, the system and heat baths are uncorrelated 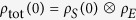. For arbitrary strength coupling between the system and environment^31^, the internal energy is defined by 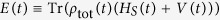, and the first law relates its changes to work and heat 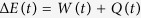 with the work 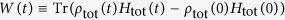 performed on the system in the time interval [0, *t*], which is also given by


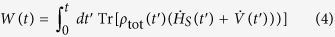


and the total heat flow 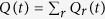 split in reservoir contributions


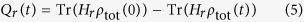


given by minus the change in energy of the *r*-reservoir.

Considering 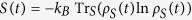 as the thermodynamic entropy of the system and 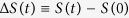, it is found that 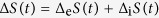 with the entropy flow 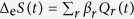 determined by the heat flows in Eq. (5) and the entropy production^31^


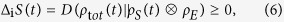


with 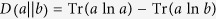. Unitarity, expressed through the invariance of 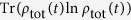 under the time evolution of the full system, plays a crucial role in the splitting of entropy change in the entropy flow and a positive entropy production. In the weak-coupling limit 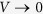 and assuming that the open system satisfies a Lindblad equation obtained from the Born-Markov-secular approximation^30^, the rate of entropy production 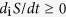 and the above expressions for work and heat take the standard form given in Eq. (3) and Eq. (2) respectively. This is shown in section methods by considering the method of full-counting statistics^43^. However, the Lindblad models investigated in^7^,^8^,^9^,^10^,^12^,^13^,^14^,^15^,^16^,^17^,^18^,^28^ are not obtained from the weak-coupling limit and do not satisfy local-detailed-balance. Thus to obtain the appropriate expressions for the thermodynamical quantities in boundary driven systems we apply in the next section the previous formulation, in particular Eqs (4),(5),(6), to a system plus environment evolving unitarily in which the reduced density matrix for the system satisfy a boundary driven Lindblad equation in an exact limit.

## The repeated interaction scheme

Let us consider a finite system with time-independent Hamiltonian *H*_*S*_ and left (*L*) and right (*R*) reservoirs composed of an infinite set of identical non-interacting finite systems with Hamiltonian 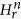, i.e., 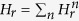, where *r* is *L* or *R*. Each 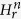 interacts with the system for a time span *τ*. This interaction is always of the same form, but to emphasize that interactions occur with different copies 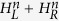 in different time intervals, we write it as 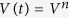 if 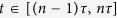 with 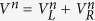. At 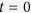, the system and reservoirs are decoupled, i.e., 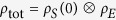, with 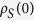 arbitrary and 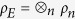, where 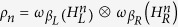. At 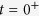, the system begins to interact with the first copy 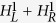, and after a lapse of time *τ*, the state of the total system is 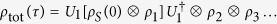 . Then, at *t* = *τ* + 0, the interaction with the first copy is replaced by an interaction with the second copy for a time *τ* and so on. A recursion relation for the state of the system is obtained^6^,^7^ by tracing out the *n*th copy of the environment (denoted as Tr_*n*_)


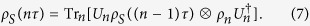


The unitaries are 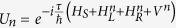. This is the repeated interaction scheme. For simplicity we considered only two heat-baths but the generalization to several reservoirs is straight forward.

Let us consider the change of thermodynamical quantities in the time intervals of length *τ*. Crucially, due to the resetting of the heat baths, the interaction term is time dependent. According to Eq. (4) for time-independent 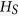, work is performed at the discrete times 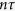 where the interaction between the system and the environment changes because the copy in interaction changes. Performing the integral in Eq. (4) between an initial time 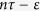 and a final time 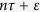, we obtain 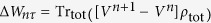 in the limit 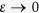. We simplify this expression with the standard^30^ assumption that 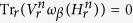. This condition will be repeatedly used; it allows us to split 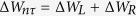 (we drop the index *nτ*) with


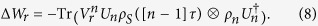


We use Tr_*r*_ to denote the trace over the *r* = *L* or *r* = *R* system and Tr to denote the full trace.

The heat flow from the bath to the system in the time interval of length *τ* where the system interacts with the *n*th copy is evaluated from Eq. (5)


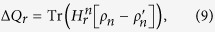


where 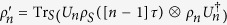 is the density matrix of the *n*th copy of the environment at the end of the interaction with the system.

The entropy production 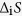 in the time lapse *τ* is obtained from Eq. (6), and after some manipulations^31^,^32^, it can be written as the sum


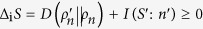


where the mutual information 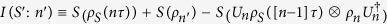 quantifies the correlations built up between the system and the *n*th copy after time *τ*. Note that 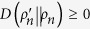 and 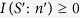 and vanishing entropy production requires 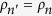 and the absence of correlations between the system and the copy 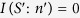. Note that because before the interaction the state of the system is arbitrary and uncorrelated with the product of thermal states of the copy, the theory of 31,32 applies independently of the correlations built between the system and previous copies.

### Heat, work and boundary-driven Lindblad equation

The index *n* is associated with the copy that interacts in the interval of time 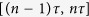, but the copies are all identical prior to the interaction (a tensor product of two canonical distributions) and the interaction 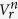 is always of the same form. Because no confusion will arise, we drop the label *n* and denote the interaction 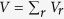, the Hamiltonian of the bath copy *H*_*r*_ and the state 
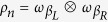
 with 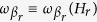. It was shown^6^,^7^ that for *V*_*r*_ that satisfies 
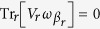
 and whose strength is scaled with *τ* as 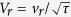, the system evolution Eq. (7) in the limit 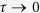 converges to a Lindblad evolution (see methods)


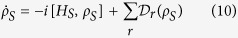


with 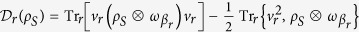. This equation applied to particular systems provides boundary-driven Lindblad equations.

Consider now 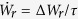 and 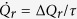 with 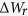 in Eq. (8) and 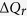 in Eq. (9). In the limit 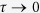 with 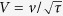, we obtain (see methods)


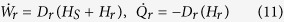


where 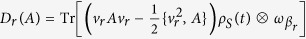. Note the first law 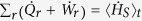, where 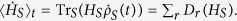 Finally, we express the entropy production rate as the difference between the time derivative of the von Neumann entropy and the entropy flow


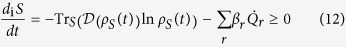


where the first term is computed using Eq. (10) with 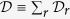 and the second term is computed from Eq. (11). Eqs (11),(12) provide appropriate thermodynamic expressions for systems evolving with Eq. (10). Now we illustrate our findings in spin 1/2 chains.

## Spin models

Consider an XY spin 1/2 chain with Hamiltonian


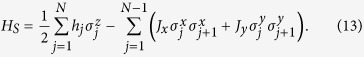


In the repeated interaction scheme we consider the couplings


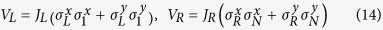


to a left *r* = *L* and a right *r* = *R* spin 1/2 reservoir copy with Hamiltonians 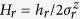, and we take *h*_*L*_ = *h*_1_ and *h*_*R*_ = *h*_*N*_. To obtain the boundary-driven Lindblad model, we scale 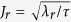. The canonical density matrices 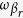 are fully characterized by the magnetization 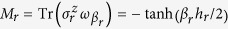.

Evaluating the second term on the right-hand side of Eq. (10) yields the dissipator in the Lindblad from 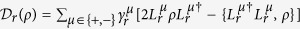 with 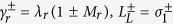 and 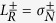 where 
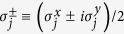
. Note that 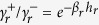.

This system does not satisfy local-detailed-balance with respect to the Gibbs state, i.e. 
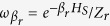
 is not the solution of 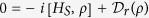 with *r* either *R* or *L* because 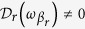. What can be shown is that these dissipators thermalize the single spin in the boundary if we disconnect it from the rest of the chain. Indeed let us consider the *L* dissipator


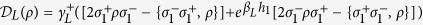


upon evaluation we see that 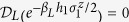. This is the generic situation in boundary driven Lindblad systems.

The expression for power and heat Eq. (11) can be evaluated using the system hamiltonian Eq. (13), the coupling Eq. (14), the bath hamiltonian 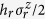 and the corresponding 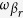. One obtain (we take 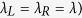



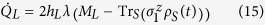


and


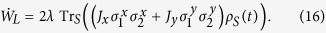


Replacing the indices {*L*, 1, 2} by 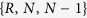 in Eqs (15),(16) one has the corresponding 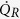 and 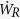. To compute this quantities, we obtain 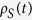 by solving the Lindblad equation^44^.

Consider the case in which the system interacts with one bath (for instance the left bath, but we drop the label *L*). In general, two situations can occur: the system relaxes to thermodynamic equilibrium in which all current vanishes or the system reaches a NESS if it is externally driven.

### XX chain coupled to one bath

**Figure 1:**
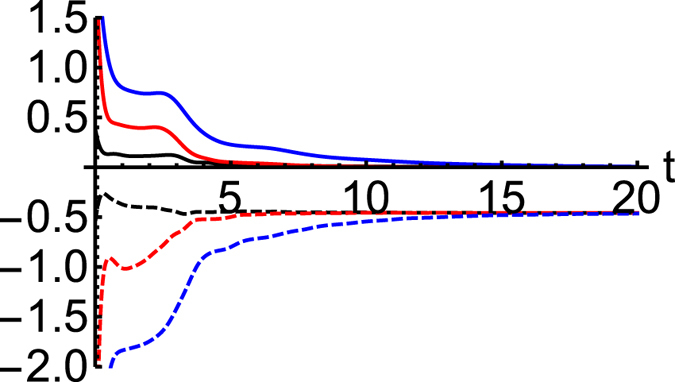
As a function of time *t* plots of 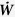 (blue), 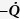 (black) and *d*_i_*S*/*dt* (red) for an XX (*J*_*x*_ = 1 = *J*_*y*_) and 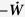 (blue, dashed), 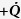 (back, dashed) and −*d*_i_*S*/*dt* (red, dashed) for an XY (*J*_*x*_ = 1 = 0.5*J*_*y*_) chain. In both cases, the chain has *N* = 5 sites with *h*_*i*_ = 1, *i* = 1,5 coupled with *λ* = 1 to a single left bath of *β* = 1 and *h* = 1.

An XX spin chain (*J*_*x*_ = *J*_*y*_) in a uniform magnetic field *h*_*i*_ = *h* coupled to a single bath relaxes to equilibrium: the entropy production rate, heat flows and power vanish. The equilibrium density matrix is not generally a canonical distribution but rather, as one can prove, is given by a generalized Gibbs state 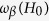 with 
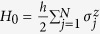
, which is a conserved quantity, i.e., 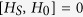. This state is a product state of the canonical density matrices *ω*_*β*_ for each spin of the chain and all equal to the one of the reservoir copy. Therefore, 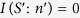 and 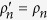, i.e., 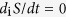. Figure 1 illustrates the relaxation to this equilibrium state by depicting the decaying power, heat flow and entropy production rate.

### XY chain coupled to a single bath

For an XY chain, we found that the system reaches a driven NESS. In this NESS, entropy production is strictly positive and constant, and because 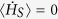, the first law gives 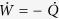. Furthermore, by combining the first and second laws, we have that 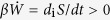 because in NESS, 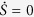. See Fig. 1.

### XX chain coupled to two baths

**Figure 2:**
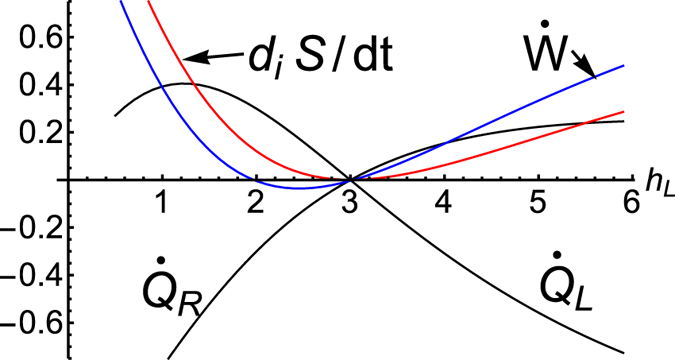
For a *N* = 5 site XX chain with *J*_*x*_ = *J*_*y*_ = 3, *h*_2_ = *h*_3_ = *h*_4_ = 5, *h*_5_ = *h*_R_ = 2, *β*_*L*_ = 0.8, *β*_*R*_ = 1.2, and *λ* = 1, we depict 
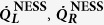
, 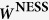 and 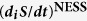 as a function of *h*_*L*_ = *h*_1_. There are two special values for *h*_*L*_. At *h*_*L*_ = 3, where 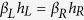, all quantities vanish (equilibrium state). At 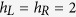, 
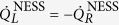
 and thus 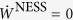 (non-driven steady state).

Consider a hot left and a cold right heat baths 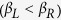 connected by an XX spin 1/2 chain with the Hamiltonian in Eq. (13) with *J*_*x*_ = *J*_*y*_ = *J*. The NESS in the special case of a uniform magnetic field was analyzed in^7^. The power and heat from the reservoir to the system are given by Eqs (15),(16). In Fig. 2, we plot 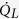, 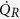, 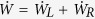 and 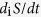 in the NESS as functions of *h*_*L*_. We observe that the heat flows can change sign and that for *h*_*R*_ = *h*_*L*_, they have opposite signs, i.e., 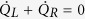, which means that 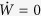. We also observe in Fig. 2 that 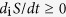 and vanishes only when 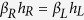, that is, the second law holds even when heat flows from cold to hot, as is the case for 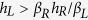, a situation that would appear to be a contradiction to the Clausius statement of the second law if we do not realize the presence of 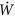.

The previous numerical study of boundary-driven spin chains can be complemented with exact results for power and heat in a two-site boundary-driven spin chain obtained from a full analytical solution of the NESS (see methods). In the NESS, the expression for power Eq. (16) and heat Eq. (15) can be written in terms of the spin current^44^


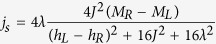


as 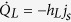, 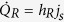 and 
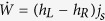
. Thus, for 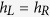, there is no power, but as the previous expression shows, this does not mean that the spin current vanishes. Moreover, the entropy production rate in the NESS is


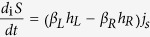


i.e., the spin current *j*_*s*_ and the affinity 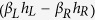 characterize the rate of entropy production in the NESS, and because 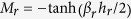, the sign of the entropy production rate is given by 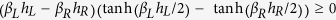, where the equality holds only if 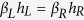. Let us end this analysis by noting that for 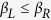, this system behaves as a heat engine for 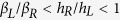 with efficiency 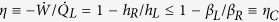, as a refrigerator for 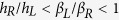 with efficiency 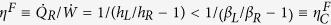 and as a heater for 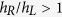. Note that the efficiencies are independent of temperature. These are steady-state operating engines analogous to those in^45^.

## Discussion

In conclusion, the repeated interaction scheme provides a physical description of a system interacting with an environment that, in an appropriate limit, provides a boundary-driven Lindblad equation for the system. The Lindblad operators that appear in this equation are determined by the interaction of the system with the environment, the Hamiltonian of the copies that form the bath and, importantly, by the fact that it is refreshed constantly. By computing the thermodynamical quantities for the full system plus the environment, one can derive the corresponding expressions for the boundary-driven model. One important observation is that due to the refreshing of the reservoir, work is done or extracted by the external agent in charge of this refreshing. This power drives the system out of equilibrium. Note that this power appears even if the system Hamiltonian and Lindblad operators are time independent. We applied our results to spin chains. In the single bath case, we found that an XX spin chain with a homogeneous magnetic field relaxes to thermal equilibrium, i.e., a state with zero entropy production, while an XY spin chain reaches a driven NESS, a state with a non-zero entropy production 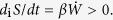 In the two heat bath case, the XX chain for different temperatures 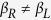 and a homogeneous magnetic field reaches a non-driven 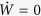 NESS and an equilibrium state for 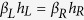 where the entropy production rate, power, heat flows and spin currents vanish. For inhomogeneous magnetic fields, the chain reaches a driven 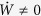 NESS. Jumping to a broader context, this work shows that the knowledge of a Lindblad equation for an open system does not determine the heat flows or other thermodynamical quantities. These quantities also depend on the properties of the environment and how the system is coupled to it. Here, we have obtained appropriate expressions for heat flows and power for interactions with an environment of a type recently implemented in a laboratory^5^. But when the reservoir is weakly and passively coupled to the system, i.e. there is no work cost in achieving the coupling, the system is appropriately described by a global^28^ Lindblad equation and the thermodynamical quantities by Eq. (2). Finally, this work is also an extension of quantum thermodynamics to a class of open quantum systems without local-detailed-balance.

## Methods

We provide here some details of the calculations mentioned in the main text.

### Work, heat and boundary-driven Lindblad equation from the repeated interaction scheme

For completeness we derive Eq. (10) and Eq. (11) of the main text. Consider 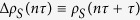 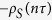. We have from Eq. (7) of the main text that


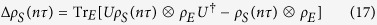


where we have dropped the label *n* from *U* and *ρ*_*n*_ in Eq. (7) because the copies are identical and the interaction 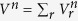 is always of the same form. The trace Tr_*n*_ over the state 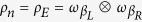 is denoted Tr_*E*_. The unitary 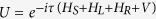 in (17) is expanded for small *τ* considering the scaling 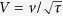 and 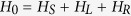



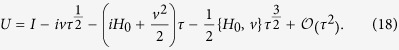


Now, because 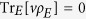 the leading order in the right hand side of (17) is 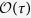. Thus, we divide by *τ* and take the limit 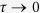 and 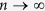 such that 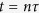 and obtain


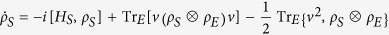


where the equality 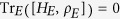 was used.

Now we use Tr_*r*_ to denote the trace over the *r* = *L* or *r* = *R* system and Tr the full trace. Because 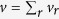 and 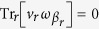, it is possible to split the last two terms in contributions for each reservoir giving Eq. (10) in the text:


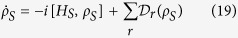


with 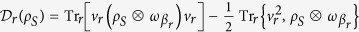.

We continue with the derivation of Eq. (11) of the main text. Let us start from 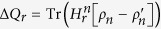, i.e. Eq. (9), where 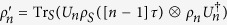. Dropping as before the label *n*, in the limit 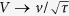 and 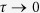 we can replace *U* by (18). The leading order of 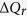 is 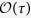



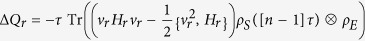


or 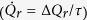



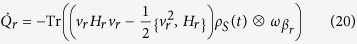


Consider Eq. (8) now i.e. 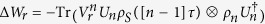. As before we drop the label *n*. The leading order is also 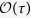 but we need *U* up to 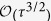 because *V* is 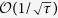, 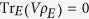 and 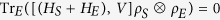. We obtain


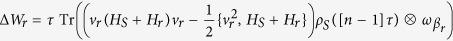


or 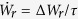



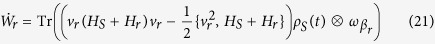


Expressions (20) and (21) correspond to those in Eq. (11) from the main text.

### The two spin XX chain with inhomogeneous magnetic field

Consider a XX two sites spin chain and the corresponding Lindblad dynamics Eq. (1) with *H*_*S*_ given by Eq. (13) main text (with *J*_*x*_ = *J*_*y*_ = *J*, *h*_1_ = *h*_*L*_ and *h*_2_ = *h*_*R*_) and the Lindblad dissipator


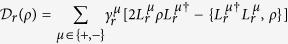


with 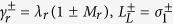 and 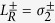 where 
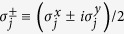
. This system is fully characterized by the correlation functions 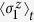, 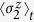, 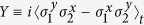 and 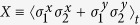 where 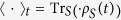. They satisfy a close system of equations:


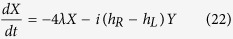



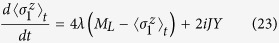



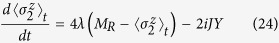



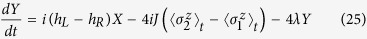


From Eqs (15,16) in the main text we note that 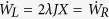, while the first term in the right hand side of (23) is 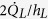 and the corresponding term in (24) is 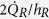. Moreover the spin current^44^ is 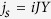. In the steady state the left-hand-side of the system (22),(23),(24),(25) vanishes and 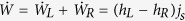, 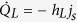 and 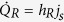. The current given in the main text is obtained by solving the full system in the NESS.

### Heat from a given reservoir in the weak-coupling limit

Consider a system coupled to several reservoirs as discussed in *“universe” under unitary evolution*. The heat that comes from one of them, for instance the *r* = *L* reservoir is 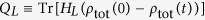. The methods developed in full counting statistics^43^ gives 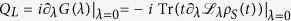 where 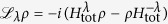 is a modified evolution super-operator with 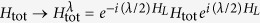. When this modification is done for a system in the weak coupling Born-Markov-secular approximation one obtain^46^,^47^ 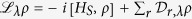 where only the dissipator associated to the *r* = *L* reservoir depends on *λ* as


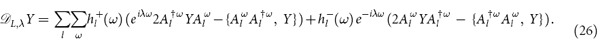


Here 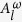 are system eigen-operators obtained from the coupling of the system to the left reservoir^30^,^46^,^47^ and 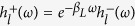. A slow time dependence of the system can be included, see^46^. From Eq. (26) we obtain


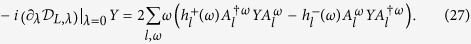


Thus 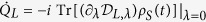 where we used that in this limit the dynamics is Markovian. We have to compare this with the heat flow defined in section *“open system weakly and passively coupled to the environment”*, 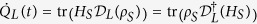, where the dissipator 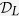 in the same weak coupling Born-Markov-secular approximation is given by 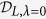, from which we compute


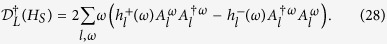


To obtain this we used 
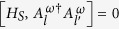
30. Taking the trace in Eq. (27) and in Eq. (28) the desired equality 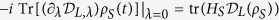 is found. Now, since the heat flow to a system weakly and passively coupled to the *L* heat-bath is given by 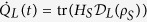, the corresponding definition for work follows and the entropy production given in Eq. (3) as well.

## Additional Information

**How to cite this article**: Barra, F. The thermodynamic cost of driving quantum systems by their boundaries. *Sci. Rep.* **5**, 14873; doi: 10.1038/srep14873 (2015).
